# The Value of Serum CA19-9 Levels in predicting Extent of Hepatic Fibrosis in Patients with Chronic Hepatitis B

**DOI:** 10.5005/jp-journals-10018-1122

**Published:** 2015-01-06

**Authors:** Mohammad Sayedul Haque, Sharmin Sultana, Mamun-Al Mahtab, Ayub Al-Mamun, Salimur Rahman

**Affiliations:** 1 Department of Hepatology, Bangabandhu Sheikh Mujib Medical University, Shahbag, Dhaka, Bangladesh; 2 Department of Virology, Bangabandhu Sheikh Mujib Medical University, Shahbag, Dhaka, Bangladesh

**Keywords:** CHB, Liver biopsy, CA19-9.

## Abstract

This cross-sectional observational study was conducted to evaluate the implication of CA19-9 level in predicting the extent of hepatic fibrosis in patients with chronic hepatitis B (CHB). Fifty treatment-naive patients with CHB were divided into three groups: group I, patients with no fibrosis (F0; n = 3); group II, patients with mild fibrosis (F1; n = 25) and group III, patients with moderate fibrosis (F3; n = 22). There was no significant (p > 0.05) difference in levels of serum bilirubin, serum alanine aminotransferase, alkaline phosphatase, and alpha fetoprotein among groups I, II and III. Also, mean CA19-9 level did not differ among the three groups. It was observed that, for group II, AUC was 42.6%, sensitivity 52%, specificity 32%, considering cut-off value as 2.8 U/ml to predict F1 liver fibrosis. For group III, AUC was 57.7%, sensitivity 68%, specificity 50% considering cut-off value as 3.2 U/ml to predict F3 fibrosis. The levels of CA19-9 level do not seem to have significant predictive implication to assess hepatic fibrosis.

**How to cite this article:**

Haque MS, Sultana S, Al-Mahtab M, Al-Mamun A, Rahman S. The Value of Serum CA19-9 Levels in predicting Extent of Hepatic Fibrosis in Patients with Chronic Hepatitis B. Euroasian J Hepato-Gastroenterol 2015;5(1):16-18.

## INTRODUCTION

Chronic hepatitis B (CHB) is the major cause of progressive liver disease that can eventually evolve into cirrhosis and its end-stage complications, including hepatic decompensation and liver cancer. Most of this disease burden occurs in developing countries, particularly in China, South-East Asia and Africa where 8% or more of the population are chronically infected with hepatitis B virus (HBV).^1^ Studies suggest that advanced fibrosis and cirrhosis develop in about 20 to 40% patients with chronic viral hepatitis.^2^ Assessment of hepatic fibrosis is, therefore, an important parameter for evaluation and follow-up of chronic viral hepatitis irrespective of etiology.

Liver biopsy has been the gold standard to evaluate the histological stage of liver fibrosis and an integral part of management of CHB. Liver biopsy is of great importance for assessing hepatic fibrosis,^3^ but is poorly suited for active monitoring, because it is invasive, expensive and is associated with morbidity. Thus, development of alternatives that are safe, inexpensive and reliable is a priority.^4^ In addition to this, liver biopsy is not feasible in all hospitals of Bangladesh due to lack of expertise, limitation of prebiopsy investigations, ultrasonography, manpower, proper nursing care, post complication management, intensive care unit facilities, and lack of bed availability for hospital admission. The limitations of liver biopsy have led to the identification of alternative possibilities to assess liver fibrosis, mainly by noninvasive methods.

CA19-9 is a tumor-associated antigen, shown to be upregulated in hepatocytes of patients with a significant increase in chronic hepatitis, fibrosis and cirrhosis, providing a marker for early detection of hepatic fibrosis. In developing countries with limited resources like Bangladesh, determination of serum CA19-9 level may be an excellent parameter to predict liver fibrosis.^5^ Moreover, the invasiveness, cost and life-threatening risk of liver biopsy can be avoided by measuring the level of serum CA19-9. In this study, measurement of serum CA19-9 was done in CHB patients to assess if the levels of serum CA19-9 and stages of hepatic fibrosis would help to minimize the need of liver biopsy in CHB patients.

## PATIENTS AND METHODS

This cross-sectional observational study was conducted in the Department of Hepatology, Bangabandhu Sheikh Mujib Medical University (BSMMU), Dhaka, Bangladesh, between July 2010 and June 2012. A total of 50 treatment-naive patients with CHB aged between 18 and 50 years of both sexes were enrolled in this study. Informed written consent was obtained from every patient. Detailed history and clinical examination was performed and documented in a structured questionnaire data collection sheet. Relevant baseline investigations including anti-HCV, CA19-9, serum alkaline phosphatase (ALP), serum bilirubin, prothrombin time, blood grouping, alpha fetoprotein (AFP), serum creatinine, chest X-ray, ultrasonography of whole abdomen and endoscopy of upper gastroenterology were done. Moreover, to diagnose CHB, serum alanine aminotransferase (ALT), serum hepatitis B surface antigen (HBsAg), hepatitis B e antigen (HBeAg) and HBV DNA were assessed. Chronic hepatitis B patients with coexistent hepatitis C virus (HCV) infections, cirrhosis of liver, decompensated liver disease (ascites, jaundice, hepatic encephalopathy), hepatocellular carcinoma, stomach cancer, abdominal lump, chronic kidney disease, positive stool occult blood test, nonalcoholic fatty liver disease (NAFLD), X-ray findings of consolidation or pleural effusion, pregnant woman and patient who did not give written consent were excluded from this study.

Liver biopsy was done if baseline prothrombin time was not >4 seconds beyond control value. After having informed written consent from the patient, liver biopsy was done using a tricut biopsy needle, G14, 15 cm in length. The specimen fixed in 10% formalin was sent to the Department of Pathology, BSMMU, for routine processing. Patients were divided into three groups according to the histopathological results (HAI and Knodell score): group I includes patients with no fibrosis (F0; n = 3), group II includes patients with mild fibrosis (F1; n = 25) and group III includes patients with moderate fibrosis (F3; n = 22).

Serum CA19-9 was assessed using the chemilu-miniscence principle (IMMULITE 2000 XPi Immunoassay Systems, Siemens, USA; ADVIA Centaur CP Immunoassay System, Siemens, USA).

## STATISTICAL ANALYSIS

All collected data were analyzed by SPSS version 16. Data were expressed as mean ± standard deviation (SD) where data were normally distributed, frequencies or in percentages until mentioned otherwise. Comparison between the groups was done by independent samples t-test. Comparison of mean values among the groups was assessed by one-way ANOVA (Hochberg’s and Games-Howell tests). Receiver operating characteristic (ROC) curve was constructed to determine the cut-off values of CA19-9 for the prediction of hepatic fibrosis at different groups. Sensitivity and specificity were expressed in percentages. p-value of <0.05 had taken significant for the study.

## RESULTS

The demographic data and grouping of patients have been shown in Table 1. Among 50 CHB patients, a male predominance was observed in this study: male 40 (80%) and female 10 (20%). Most of the patients of group I were comparatively younger (22.67 ± 3.78 years), while for groups II and III were older (26.16 ± 5.98 years and 25.81 ± 5 years). There was no significant difference in serum bili-rubin, ALT, AFP values among the three groups (Table 1). There was no significant difference of CA19-9 levels in group I patients (3.70 ± 1.05 U/ml) compared to patients of groups II and III (5.54 ± 5.19 U/ml and 6.01 ± 4.73 U/ml respectively (Table 1).

Multiple comparisons of CA19-9 values among the different groups were assessed by one-way ANOVA test and found liver fibrosis values cannot be predicted by CA19-9 value.

In the current study, it was observed that, for group II, AUC was 42.6%, sensitivity 52%, specificity 32% considering cut-off value as 2.8 U/ml to predict F1 liver fibrosis. For group III, AUC was 57.7%, sensitivity 68%, specificity 50% considering cut-off value as 3.2 U/ml to predict F3 fibrosis (Table 2).

**Table 1:** **Table 1:** Demographic details and laboratory findings

		*Group I*		*Group II*		*Group III*	
Fibrosis (no. of patients)		F0 (n = 3)		F1 (n = 25)		F3 (n = 22)	
Age (years)		22.67 ± 3.78		26.16 ± 5.98		25.81 ± 5.83	
M:F		1:2		4:1		3.3:1	
S. bilirubin (mg/dl)		0.49 ± 0.12		0.67 ± 0.19		0.66 ± 0.19	
SGPT (U/L)		46.34 ± 14.36		40.24 ± 1.26		42.14 ± 1.22	
ALP (U/L)		51		81.64 ± 3.6		85.54 ± 2.46	
AFP (ng/ml)		2.74 ± 1.19		3.9 ± 3.0		3.9 ± 3.0	
CA 19-9 (U/ml)		3.70 ± 1.05		5.54 ± 5.19		6.01 ± 4.73	

**Table 2:** **Table 2:** CA19-9 value for the prediction of F1 and F3 liver fibrosis

*Groups*		*AUC*		*Cut-off value* *(U/ml)*		*Sensitivity* *(%)*		*Specificity* *(%)*	
II		0.426		2.8		52		32	
III		0.577		3.2		68		50	

## DISCUSSION

Liver fibrosis is a progressive process with different features, prognoses and predictors of death. Increased CA19-9 level significantly correlates with the stages of fibrosis and reflects as the noninvasive marker for advanced liver fibrosis. So, measurement of serum CA19-9 level can be useful for the prediction of hepatic fibrosis in CHB patients. There was no significant (p > 0.05) difference in laboratory parameters (serum bilirubin, ALT, ALP, AFP) among three groups.

Among the 50 patients, F0 (stage 0) fibrosis was 3 (6%), F1 (stage 1) fibrosis was 25 (50%) and F3 (stage 3) fibrosis was 22 (44%). No patients in stage 2 or stage 4 because the histopathological examination were done in hematoxylin and eosin stain in which F2 (stage 2) fibrosis could not be demarcated. As cirrhosis was clinically excluded from the study, so F4 (stage 4) fibrosis was absent in this study.

In the current study, we found that in patients with chronic HBV infection, liver fibrosis prediction by serum CA19-9 level was not feasible in the prediction of F1 and F3 fibrosis (AUROC = 42.6% and 57.7%). It has been reported a highly significant positive correlation between serum level of CA19-9 and the stage of liver fibrosis (p < 0.01).^6^ As regards CA19-9 in differentiating the presence (F1) from absence (F0) of liver fibrosis, they found that the best cut-off value was 2.98 U/ml with a sensitivity of 86.7%, specificity of 60%. Furthermore, the best cut-off value in predicting severe liver fibrosis (F4) was 33.87 U/ml with a sensitivity of 93.8%, specificity of 88.2% in that study. In the current study, we found for group II AUC was 42.6%, sensitivity 52%, specificity 32% considering cut-off value as 2.8 U/ml to predict F1 liver fibrosis. For group III, AUC was 57.7%, sensitivity 68%, specificity 50% considering cut-off value as 3.2 U/ml to predict F3 fibrosis. Multiple comparison by one-way ANOVA showed that mean CA19-9 value does not differ among the three groups. The observed difference of our study with that of other studies^6,7^ may be due to that we have studied patients with CHB but they analyzed patients with chronic hepatitis C.
